# Epidemic Causes

**Published:** 1854-08

**Authors:** 


*Epidemic Causes.*—We find it no easy matter, in these times of constant occupation, to keep up the editorial department of the Journal. One feels little like sitting down quietly to write, after a fatiguing or exciting day's labor. Even with all the spare time necessary, it is difficult to condense ones thoughts, and place them in a readable and interesting shape.

But we must fill up our vacant space, and we cannot do better at this time, than to trench a little upon the matter of an article we hope to prepare for our September number, relative to the epidemic constitution of the atmosphere.

On reference to other pages in the editorial department, it will be seen that during the month of June the dew-point was generally low until the latter days of the month.

On the 18th, after averaging below 60° for a week, it continued high until the 30th. During this period some isolated cases of cholera occurred, as follows: 23d, 1 case; 25th, 1; 26th, 3; 28th, 1; 29th, 2; and 30th, 1. Of these 9 cases, 6 were fatal.

During the week ending July 1st, the average dew-point at 2, P. M., was 63°; the dew-point ranging lower at that hour than at any other in the day. On July 1st, there were 4 cases, of which 3 were fatal.

During the week ending July 8th, the dew-point averaged 64° at 2, P. M., and 27 cases occurred in the same period, of which 19 died.

During the second week of July, ending on the 15th, the average dew-point at 2, P. M., was 58°; the number of cases of cholera was 42, of which only 19 died.

During the third week of July, ending on the 22, the average dew-point was 69° at 2, P. M. The number of cases occurring within the city limits, excluding the cases at the Poor House, was about 54; of which 32 died.

At this time we consider the epidemic established, and though we have not had as yet a great number of cases, yet sundry circumstances connected with sanitary hygiene enable us to derive from this number of cases, and the contrast they afford to other localities in the same atmospheric condition, some very important thoughts relative to the causation of cholera.

The sanitary condition of Buffalo this season is, owing to the efficient action of our Board of Health, very good. The streets are clean; low and wet places were drained early in the season; houses most exposed to cholera causes have been shut up or torn down, and, so far as possible, no causes of cholera were left, except those dependent on the amount of humidity in the atmosphere from general causes. Local causes of humidity were obviated.

In this comparison of dew-point with cholera, if we draw two lines on a graduated scale, the dew-point will run parallel to the deaths. In the second week we had an apparent increase of cases, (from 27 in the first week, to 42 in the second,) but many of these cases originated during the first week, and we find that the number of deaths was no greater than in the first week.

In the third week, the extremely high dew-point was accompanied, not only by an increase in the cases, but by a corresponding fatality—32 dying out of 54 cases. As we mentioned before, this does not include the Poor House mortality, on which we propose to bestow a little time.

Some cases of cholera had occurred, from time to time, without great fatality, until the 18th, at evening; (dew-point, at 2, P. M., 65° 33, and at 7, P. M., 70° 33) it broke out in the isolated building containing the insane, 53 in number. On the evening of the 19th, (dew-point at 2, P. M., 72° 33) 15 were dead out of about 20 attacked, and of those living four were in collapse.

Now although we can trace the direct influence of the dew-point in this mortality, yet it is evident that some other causes are to be sought, inasmuch as the number of deaths in this city on that day was only 4. These causes are to be found in another part of this issue, in an article written for the Commercial Advertiser. It is unnecessary for us to dwell here on these causes. Suffice it to say that a deep feeling was roused in community, and our Board of Health have interfered in this matter with some strong measures, which we hope may be fully carried out. The infamy which attaches to this epidemic should not be charged upon the city of Buffalo. Though mostly supporting the institution, it has but a minority influence in its control.

On this same fatal 18th and 19th of July, the cholera appeared at Suspension Bridge in a most malignant form. The most disastrous days were on Saturday (dew-point 72° 33,) and on Sunday (dew-point 69° 33). The epidemic continued in an unmitigated form until the 26th, when the dew-point dropping to 58°, no new cases occurred, and a tendency to convalescence was manifested.

The local causes, predetermining this epidemic, were very evident, and will be the subject of full discussion in a future number. H.

---

We have had the pleasure of examining a bedstead for invalids, at Mr. A. Cooley's Furniture Ware Rooms, 115 Main Street, Buffalo. We think the invention a very ingenious and useful one, particularly as its price can be brought within the limits of moderate means.

It answers all the indications desirable as to position, coolness, and ease of changing the linen. We append a note from Dr. Hamilton, in which we concur. H.

Having to-day examined "Van Allen's Invalid Bedstead," at the request of Mr. Cooley, I take pleasure in saying that it is the most convenient and ingenious bedstead for invalids that I have seen.

FRANK H. HAMILTON.

Buffalo, July 14, 1854.

